# Safety and effectiveness of standardized exercise training in patients with pulmonary hypertension associated with heart failure with preserved ejection fraction (TRAIN-HFpEF-PH): study protocol for a randomized controlled multicenter trial

**DOI:** 10.1186/s13063-023-07297-x

**Published:** 2023-04-19

**Authors:** Eglė Palevičiūtė, Jelena Čelutkienė, Toma Šimbelytė, Lina Gumbienė, Elena Jurevičienė, Diana Zakarkaitė, Sigitas Čėsna, Christina A. Eichstaedt, Nicola Benjamin, Ekkehard Grünig

**Affiliations:** 1 Clinic of Cardiac and Vascular Diseases, Institute of Clinical Medicine, Faculty of Medicine, Vilnius University, Santariskiu-2, 08661 Vilnius, Lithuania; 2 Faculty of Medicine, Vilnius University, Vilnius, Lithuania; 3 Centre for Pulmonary Hypertension, German Center for Lung Research (DZL), Thoraxklinik Heidelberg gGmbH at Heidelberg University Hospital, Translational Lung Research Center Heidelberg (TLRC), Heidelberg, Germany; 4 Laboratory for Molecular Genetic Diagnostics, Institute of Human Genetics, Heidelberg University, Heidelberg, Germany

**Keywords:** Exercise training, Heart failure with preserved ejection fraction, Pulmonary hypertension, Study protocol, Randomized controlled trial

## Abstract

**Background:**

Left heart failure (HF) is characterized by an elevation in left-sided filling pressures, causing symptoms of dyspnea, impairing exercise capacity, and leading to pulmonary venous congestion and secondary pulmonary hypertension (PH). There is an increased incidence of PH associated with left heart disease, particularly with heart failure with preserved ejection fraction (HFpEF-PH). Treatment possibilities in HFpEF-PH are non-specific and very limited, thus additional pharmacological and non-pharmacological therapeutic strategies are needed. Various types of exercise-based rehabilitation programs have been shown to improve exercise capacity and quality of life (QoL) of HF and PH patients. However, no study focused on exercise training in the population of HFpEF-PH. This study is designed to investigate whether a standardized low-intensity exercise and respiratory training program is safe and may improve exercise capacity, QoL, hemodynamics, diastolic function, and biomarkers in patients with HFpEF-PH.

**Methods:**

A total of 90 stable patients with HFpEF-PH (World Health Organization functional class II–IV) will be randomized (1:1) to receive a 15-week specialized low-intensity rehabilitation program, including exercise and respiratory therapy and mental gait training, with an in-hospital start, or standard care alone. The primary endpoint of the study is a change in 6-min walk test distance; secondary endpoints are changes in peak exercise oxygen uptake, QoL, echocardiographic parameters, prognostic biomarkers, and safety parameters.

**Discussion:**

To date, no study has investigated the safety and efficacy of exercising specifically in the HFpEF-PH population. We believe that a randomized controlled multicenter trial, which protocol we are sharing in this article, will add important knowledge about the potential utility of a specialized low-intensity exercise and respiratory training program for HFpEF-PH and will be valuable in finding optimal treatment strategies for these patients.

**Trial registration:**

ClinicalTrials.gov NCT05464238. July 19, 2022.

**Supplementary Information:**

The online version contains supplementary material available at 10.1186/s13063-023-07297-x.

## Background

Pulmonary hypertension (PH) associated with left heart disease (LHD-PH) is a growing health problem with high morbidity and mortality [1–3]. Among the various PH groups, LHD-PH is the most common form and accounts for 50–80% of all cases [1, 4–8]. The estimated prevalence of PH in HF varies depending on the method of pulmonary arterial pressure (PAP) measurement, the definition of PH, the populations studied, and the type of HF. In heart failure with reduced ejection fraction (HFrEF), PH frequency is 40–75%, as assessed by right heart catheterization (RHC) [6, 9–11]. The precise prevalence of PH associated with heart failure with preserved ejection fraction (HFpEF-PH) is still unclear. Most of the data are based on echocardiographic signs, where PH prevalence in HFpEF ranges between 52 and 83% [12, 13]. In the invasive hemodynamic assessment study, among 618 HFpEF patients, 355 (57.4%) were diagnosed with PH, and 75 (21.1%) of them had combined post- and pre-capillary PH (CpcPH) [11].

Any type of left HF is characterized by an elevation in left-sided filling pressures, causing symptoms of dyspnea [14], impairing exercise capacity [15], and leading to pulmonary venous congestion and secondary post-capillary PH. Across the spectrum of LHD, increased PAP and pulmonary vascular resistance (PVR) are related to higher disease burden and worse outcomes [16–18].

Targeted medication therapy for pulmonary arterial hypertension (PAH) and chronic thromboembolic pulmonary hypertension (CTEPH), including drugs interfering with the endothelin, nitric oxide, and prostacyclin pathways, has evolved progressively in the last decades, with increasing evidence for efficacy [1, 19, 20]. However, these drugs are not recommended in patients with LHD-PH [1, 3]. Conventional HF treatments (medical/interventional) currently remain the only therapies for these patients [1, 4, 21]. Although HF syndrome covers both, HFrEF and HFpEF, these disorders have some differences in pathophysiology, clinical characteristics, hemodynamics, cardiopulmonary interaction, and response to therapy [22–24]. While pharmacological treatments are well-established in HFrEF, the same strategies could not be convincingly proven to be effective in HFpEF [21] with the recent exception of sodium-glucose cotransporter-2 (SGLT2) inhibitors case [25, 26].

In patients with HFpEF and HFpEF-PH medication therapy options remain limited—SGLT2 inhibitors, diuretics, and treatment of comorbidities are recommended [1, 21, 25, 26]. Therefore, there is a high need for additional therapeutic strategies to improve symptoms, exercise capacity, health-related quality of life (QoL), and prognosis in these patients.

The ESC/ERS 2022 guidelines recommend complex treatment of PAH, consisting of disease-targeted and supportive medical therapy, together with non-pharmacological general measures, including supervised exercise training [1], but there are no evidence-based recommendations for the exercise therapy of LHD-PH.

Different exercise-based rehabilitation programs have already been shown to improve the physical capacity and health-related QoL of PH patients [27–33]. Most of the previous trials enrolled PAH and CTEPH patients only, excluding post-capillary PH, which is pathognomonic for LHD-PH. Standardized low-intensity exercise and respiratory training programs also demonstrated an increase in exercise capacity, lower limb muscle strength, health-related QoL, and hemodynamics in PAH and CTEPH patients; LHD-PH was an exclusion criterion in all previous studies [30, 31, 33–36].

Properly designed exercise interventions for HF patients demonstrated improvement in physical capacity, health-related QoL, and risk reduction of all-cause and HF hospitalizations [37–42]. Although HFrEF patients were predominant in the largest HF exercise training trials, several systematic reviews and meta-analyses have focused specifically on HFpEF, and confirmed the positive impact of exercising on functional capacity in HFpEF, as indicated by improved peak oxygen uptake (VO_2_peak), 6-min walk test distance (6-MWD), and health-related QoL [42–47]. The number of HFpEF-PH patients in previous HFpEF exercise training trials remains unclear [48], and none of the previous studies focused specifically on HFpEF-PH.

This study is sought to investigate whether a standardized exercise training program is safe and tolerable and may improve exercise capacity, quality of life, hemodynamics, diastolic dysfunction, and biomarkers in patients with HFpEF-PH. We used the SPIRIT Checklist when writing our manuscript [49].

## Methods/design

### Study design

This study is designed as a prospective, randomized (1:1 randomization), controlled, parallel-group, superiority, multicenter trial in patients with invasively confirmed HFpEF-PH.

The total duration of the study is planned for 3 years (Q1 2023 to Q4 2025). The follow-up of individual subjects will be performed until the last patient’s last visit. The study is registered on ClinicalTrials.gov (identifier—NCT05464238). A summary of the protocol is provided in Table 1.

**Table 1:** The World Health Organization trial registration data set: structured summary

Data category	Information
Primary registry and trial identifying number	ClinicalTrials.gov Identifier: NCT05464238
Date of registration in primary registry	July 19, 2022
Secondary identifying numbers	2019-04ET
Source(s) of monetary or material support	Heidelberg University
Primary sponsor	Heidelberg University
Secondary sponsor(s)	N/ A
Contact for public and scientific queries	Ekkehard Grünig, MD ekkehard.gruenig@med.uni-heidelberg.de Nicola Benjamin, MSc nicola.benjamin@med.uni-heidelberg.de
Public title	Safety and effectiveness of standardized exercise training in patients with pulmonary hypertension associated with heart failure with preserved ejection fraction
Scientific title	Implementation, safety, tolerability, and effect of exercise and respiratory training on 6-min walking distance in patients with pulmonary hypertension and heart failure with preserved ejection fraction (HFpEF): a randomized controlled multicenter trial in European countries
Countries of recruitment	Germany, Lithuania
Health condition(s) or problem(s) studied	Efficacy and safety of exercise and respiratory training for patients diagnosed with pulmonary hypertension associated with heart failure with preserved ejection fraction (HFpEF)
Intervention(s)	Active Comparator: Exercise rehabilitation Placebo Comparator: Standard treatment (waiting group)
Key inclusion and exclusion criteria	**Inclusion criteria:**
	- Female and male patients ≥ 18 years
	- WHO/NYHA functional classes II–IV
	- PH with HFpEF diagnosed by right heart catheterization
	- Left ventricular ejection fraction ≥ 50%
	- Patients receiving optimized therapy including intensified treatment with diuretics and who have been stable for 1 month before entering the study
	- Except for diuretics, medical treatment should not be changed during the study period
	- Able to understand and willing to sign the informed consent form
	**Exclusion criteria:**
	- Pre-capillary pulmonary hypertension
	- Congenital or acquired severe valvular diseases
	- Walking disability
	- Subject who participates in an interventional study during the course of this study
	- Severe lung disease
	- Active myocarditis, unstable angina pectoris, exercise-induced ventricular arrhythmias
	- Hemoglobin concentration less than 75% of the lower limit of normal
	- Systolic blood pressure < 85 mmHg
	- Pregnancy or lactation
	- History or suspicion of inability to cooperate adequately
Study type	Interventional, multicenter trial; intervention model description: randomized, controlled, parallel-group with crossover of the control group; masking: single (outcomes assessor); primary purpose: treatment
Date of first enrolment (estimated)	February 1, 2023
Target sample size	90
Recruitment status	Not yet recruiting
Primary outcome(s)	Changes in 6-min walking distance (time frame: baseline to 15 weeks)
Key secondary outcomes	Changes in peak exercise oxygen uptake, quality of life, echocardiographic parameters, natriuretic peptides (time frame: baseline to 15 weeks)

### Randomization and blinding

The distribution of patients to the different groups will be performed by block randomization. Group allocation will be provided in sealed envelopes, which will be prepared for each participating center by the study coordinator at Heidelberg University. Blocks for block randomization will be medium size to allow a balanced randomization sequence over time. Sites that only include a very small amount of patients may lead to a minimally unbalanced patient number. Block sizes will be the same for all centers, leading to several randomization/allocation blocks in high recruiting centers. Upon inclusion, the participants will be randomly designated to the intervention (training) or conventional (control) group. Allocation concealment will be ensured, as envelopes will not be unsealed until the patient has been recruited into the trial.

The participation in this rehabilitation program is voluntary. Participants may terminate their participation at any time.

According to the eligibility criteria, the patient should be receiving optimized and ≥ 1 month of stable treatment before inclusion in the study. Thus, we expect that the need for medication changes during the 15-week follow-up period will be minimal. However, the dosage of diuretics is permitted to be changed, and other pharmacological and non-pharmacological treatments will not be prohibited, if the treating physicians decide this to be clinically necessary. Participation in another interventional study is prohibited during the 15-week follow-up period.

Cardiologists, pulmonologists, and PH experts, blinded to the patients’ allocation groups, will perform and interpret examination tests (6-min walking test, cardiopulmonary exercise test, echocardiography, right heart catheterization, cardiac magnetic resonance imaging). Study data analytics will also be blinded to the patients’ allocation groups until the entire analysis has been completed.

### Population sampling

Candidate patients will be selected from European pulmonary hypertension centers (a list of study sites can be found at ClinicalTrials.gov), which will be continuously updated. To achieve an adequate number of enrolled participants, healthcare professionals at involved European PH centers, as well as national associations of PH patients, will be informed about the ongoing study and the opportunity to take part in it. The diagnosis of HFpEF-PH will be established according to the ESC/ERS guidelines [1, 3, 21]. Patients will be screened before their baseline visit regarding the inclusion and exclusion criteria (days − 28 to − 1) (Table 2). After 15 weeks, patients of the control group will be offered to participate in the training program (waiting-group design). All patients will visit the clinic for pre-study screening on days − 28 to − 1 (visit [V] 1), at baseline (BL) at day 1 (V2), and week 15 (V4).

**Table 2:** Patient eligibility criteria

Inclusion criteria	Exclusion criteria
1. Female and male patients ≥ 18 years 2. WHO functional classes II–IV 3. PH confirmed by RHC, showing *mPAP* > *20 mmHg at rest*; HF confirmed by invasive measurements, when *mPAWP* ≥ *15 mmHg* or *LVEDP* ≥ *16 mmHg* at rest and/or *PAWP* ≥ *25 mmHg* during exercise and/or *PAWP* > *18 mmHg* after the fluid challenge test 4. Preserved left ventricular ejection fraction > 50% *(measured by echocardiography or MRI)* 5. A patient received optimized and for ≥ 1 month stable therapy, including adequate treatment with diuretics 6. Except for diuretics, medical treatment should not be expected to change during the study period 7. Able to understand and willing to sign the informed consent form	1. Pre-capillary pulmonary hypertension *(group I, group III, group IV, group V, according to the ESC/ERS PH guidelines)* 2. Congenital or acquired severe valvular diseases *(severe aortic stenosis or insufficiency, severe mitral valve stenosis or insufficiency)* 3. Active myocarditis, unstable angina pectoris, exercise-induced ventricular arrhythmias, active liver disease, porphyria, or elevations of serum transaminases > 3 × ULN (upper limit of normal) or bilirubin > 1.5 × ULN 4. Severe lung disease: FEV1/FVC < 0.5 and total lung capacity < 60% of the normal value 5. A subject who participates in an interventional study during this study 6. Walking disability or other orthopedic limitations to exercise 7. Hemoglobin concentration less than 75% of the lower limit of normal 8. Systolic blood pressure < 85 mmHg 9. Pregnancy or lactation 10. History or suspicion of inability to cooperate adequately

*FEV1* forced expiratory volume in the first second, *FVC* forced vital capacity, *LVEDP* left ventricular end-diastolic pressure, *mPAP* mean pulmonary arterial pressure, *mPAWP* mean pulmonary arterial wedge pressure, *PH* pulmonary hypertension, *RHC* right heart catheterization, *WHO* World Health Organization

### Interventions

#### Training

The training will be based on the specialized low-intensity rehabilitation program, including exercise and respiratory therapy and mental gait training, which has been previously described [32, 35, 50, 51].

The initial phase of exercise training will be closely monitored, as it will be held in hospital for approximately 3 weeks period and later will be continued for further 12 weeks at home. In-hospital stays will be arranged country-specific, and hospitalization times may range. The rehabilitation program will comprise interval ergometer training (20 min 5 days per week), dumbbell training of single muscle groups (30 min 5 days a week), respiratory therapy (30 min 5 days per week), mental training and guided walks (individually, at least two times a week), and conventional elements, such as massages, relaxation, lectures, and patient education.

Individual training adjustment will be performed according to clinical assessments at baseline and follow-up and the patient’s subjective perception throughout the program. Training intensity will be started at 40–50% of the maximal patient’s workload, reached during the cardiopulmonary exercise test at baseline, and will be increased within the in-hospital period, trying to achieve the maximal baseline workload (maintaining 60–80% of the peak heart rate and avoiding desaturation < 90%). Overexertion must be strictly avoided. In order to ensure safety and adherence, every workout will be supervised by a physiotherapist during an in-hospital phase. Before leaving the hospital, each patient will receive individualized written recommendations on how to continue exercising at home. The patient will be asked to document daily trainings (intensity, duration, pulse rate, blood pressure, adverse events), which will be discussed at the follow-up visits.

#### Control

Patients in the control group will not receive exercise training and will continue their regular treatment and daily activities for 15 weeks. After 15 weeks, control group patients will be offered to take part in the exercise training program (waiting-group design). In this case, the final assessment of the patient in the control group will also serve as a baseline assessment for a participant in the training group.

### Individual withdrawal criteria

The patient will be withdrawn from the study at any time, if he/she wishes to discontinue or if one of the following occurs due to exercise training:

- ➢Signs of acute heart failure
- ➢Syncope
- ➢Symptomatic ventricular tachycardia
- ➢Worsening of WHO functional class

### Data collection and management

Each center will receive study-specific case record forms (CRF) to collect the data and enhance data integrity. Information obtained will be pseudonymized. The rules of medical confidentiality and data protection will be followed.

The data will be prospectively collected in every participating center. All findings including clinical and laboratory data will be documented in the patient’s medical record. The center’s principal investigator will be responsible for the patients’ safety, study conduct, and data quality, ensuring that all sections of the database are completed correctly and entries can be verified against source data. The biometrician and trial coordinator, appointed by Thoraxklinik at Heidelberg University Hospital (Core center of the study), will be able to access all (e)CRFs and check for reliability, consistency, and completeness of the data. Based on these checks, queries will be produced combined with the queries generated by visual control. All missing data or inconsistencies will be reported back to the respective recruiting center and will be followed upon completion.

### Assessments

#### Efficacy assessment

Efficacy parameters in both groups will be assessed at baseline and after 15 weeks (mean or median changes from baseline to 15 weeks, comparing changes within trained and control groups as well as mean/median changes between these groups). Patients in the training group will additionally perform the 6-MWD at the end of in-hospital rehabilitation.

The primary endpoint is to determine the effect of standardized exercise and respiratory training on exercise capacity, measured by the mean change of 6-MWD (baseline vs 15 weeks vs control group) in the HFpEF-PH population.

Secondary endpoints include mean/median changes from baseline to 15 weeks of echocardiography parameters, cardiopulmonary exercise testing, World Health Organization (WHO) functional class, measurement of safety parameters, health-related quality of life assessment (SF-36 questionnaire), anxiety and depression assessment (HADS scale), optional right heart catheterization at rest and during exercise, and optional cardiac magnetic resonance imaging (MRI). A detailed description of the evaluative parameters of each test is provided in the following paragraph (the “Procedures” section).

A follow-up interview will be performed by phone at the end of the study (last patient, last visit) to assess safety and compliance and observe survival. The long-term compliance and risk profile of exercise training will be explored to identify risk factors and record adverse events.

**Fig. 1:**
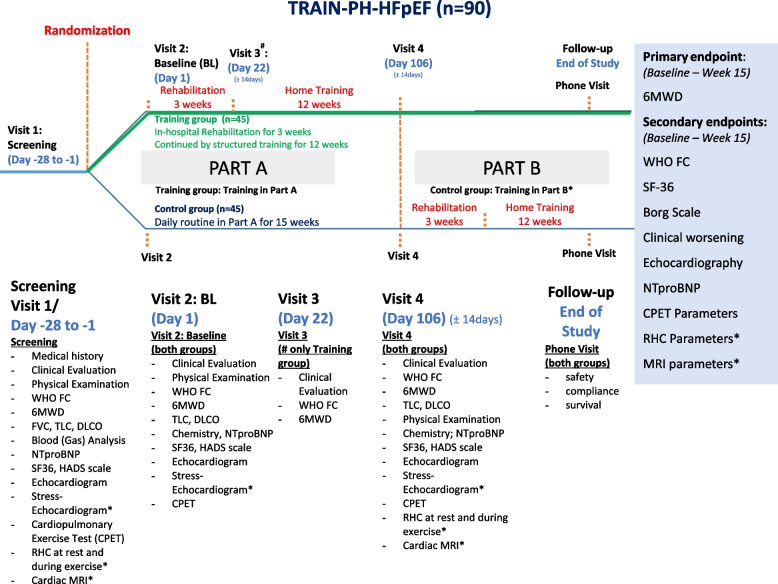
The detailed timeline of the study

The detailed timeline of the study is shown in Fig. 1.

### Procedures

#### Six-minute walking distance (6-MWD) and Borg scale

Blinded to the patients’ allocation groups, investigators will perform a 6-MWD test, according to the ATS guidelines [52]. Before and after the 6-MWD test, heart rate, blood pressure, and oxygen saturation will be measured. If the patient receives supplemental oxygen, the amount of oxygen has to be the same for each test. After the test, 6-MWD and subjective perception of dyspnea, measured by the Borg scale of 10 points, will be determined.

#### Cardiopulmonary exercise testing (CPET)

Cardiopulmonary exercise testing will be performed to assess peak oxygen uptake (peak VO_2_), peak VO_2_/kg, peak workload, heart rate, blood pressure, oxygen saturation, ventilation, ventilatory equivalent for carbon dioxide (VE/VCO_2_), VO_2_, and VCO_2_ at the anaerobic threshold. Investigators will be blinded to the patients’ allocation group.

#### Echocardiography

Investigators, blinded to the patients’ allocation groups, will perform echocardiography. Two-dimensional, Doppler echocardiography, and three-dimensional (3D) measurements will be conducted. Echocardiography measurements will include sizes of chambers, LV pump function (LV ejection fraction, determined by Simpson biplane method and 3D LV EF), LV diastolic function (*E*/*e*′ mean), RV pump function (RV fractional area change (FAC) and 3D RV EF), estimated systolic pulmonary arterial pressure (sPAP), tricuspid annular plane systolic excursion (TAPSE), tricuspid annular peak systolic velocity (TAPSV), global longitudinal RV and LV strain, and size and collapsibility of the inferior vena cava (IVC).sPAP will be estimated by the maximal velocity of tricuspid regurgitation using the Bernoulli formula. Right atrial pressure will be evaluated under consideration of IVC: 3 mmHg if the vena cava < 21 mm (width) and collapses during inspiration > 50%, 8 mmHg if the vena cava is > 21 mm and collapses during inspiration > 50%, and 15 mmHg if the vena cava is > 21 mm and collapses < 50%.

#### Invasive hemodynamic assessment (optional)

Hemodynamic parameters will be determined by right heart catheterization according to the current guidelines. Invasively measured parameters will include right atrial pressure, mean pulmonary arterial pressure (mPAP), and pulmonary artery wedge pressure (PAWP). Cardiac output (CO) will be estimated by the thermodilution method. Pulmonary vascular resistance (PVR) and cardiac index (CI) will be calculated. The values will be based on two to three measurements at one time point. Hemodynamics will be measured at rest and optionally during exercise with a supine bicycle ergometer. Investigators will be blinded to the patients’ allocation group.

#### Cardiac MRI (optional)

Investigators who will be blinded to the clinical data and group assignment will evaluate cardiac MRI. MRI measurements will include LV and RV mass, area, volumes and function (regional and global), diastolic function assessment (mitral-pulmonary flows), atrial size and function (left atrial strain and strain rate), T1 mapping/extracellular volume, and the pulmonary artery to aorta ratio.

#### Laboratory parameters

The N-terminal prohormone of brain natriuretic peptide (NTproBNP) will be assessed at baseline and after the 15-week follow-up period. Routine laboratory parameters determined according to the standards of the clinic (hemoglobin, glucose, creatinine, potassium, iron) will be collected as safety parameters.

#### Patient-reported outcomes

Quality of life will be assessed with the 36-Item Short Form Health Survey (SF-36) [53]. The questionnaire includes two main scores with physical component and emotional component scales and eight subscales. A higher score indicates a better QoL. Anxiety and depression will be assessed using the Hospital Anxiety and Depression Scale (HADS) [54]. The HADS comprises seven questions for anxiety and seven questions for depression. Scores of less than 7 indicate non-anxiety or non-depression. The higher the score, the more pronounced anxiety or depression is. Both questionnaires will be in the respective language of the country.

#### Risks assessment

Adverse reactions/adverse events and serious adverse reactions/serious adverse events will be documented throughout the trial.

### Statistical considerations and analyses

#### Sample size calculation

To estimate the effect of exercise training on 6-MWD, 90 patients are expected to be enrolled, who either receive exercise training or continue their daily lifestyle for 15 weeks. The sample size was calculated by the G*Power 3.1 program according to the results of previous studies on 6-MWD. Based on previous data and the inclusion criteria, we expect a clinically significant mean increase of 35 m with a standard deviation of the difference of 50 m. If the true treatment effect is a difference of at least 35 m with an equal standard deviation of the difference of 50 m, the two-sided Student’s *t*-test at an alpha level of 0.05 has a power of 85%, if 38 patients for each group are included. To account for a possible 15% dropout rate, we will include 45 patients in each group—90 patients in total.

#### Statistical analysis

The data from all centers will be pooled. If sample sizes allow, center effects on the primary and secondary outcomes will be analyzed.

Descriptive statistics will be displayed by treatment and control groups corrected for the active treatment group, including the usual location and scale statistics (mean, median, standard deviation, standard error, first and third quartiles, minimum, and maximum) and 95% confidence limits of the mean and median. Frequency tables for qualitative data will be provided. As an unbalanced distribution of baseline characteristics may not be completely excluded, baseline clinical parameters including patient risks and prognostic implications will be analyzed and compared between the groups.

The primary efficacy analysis will be based on an analysis of variance with the baseline values as a covariate (ANCOVA). This analysis provides a power advantage over the standard two-sided Student’s *t*-test leading to a statistical power of 85% or more. If the preconditions for ANCOVA are not met, a *t*-test with robust variance estimation (Welch test) will be used. Secondary endpoints will be analyzed by comparison of the mean changes between baseline and 15 weeks in the two groups (training vs control group) adjusted for the baseline value (ANCOVA or Wilcoxon rank sum test for non-parametric data). Categorical data will be analyzed by the McNemar test or chi-square test as appropriate. Exploratory data analysis of follow-up will be conducted by descriptive statistics and comparing baseline values and follow-up examinations. In case of ≤ 20% of missing values of a variable, a multiple imputation strategy will be used and analyzed as sensitivity analysis for data interpretation. The primary analysis set is the intention-to-treat analysis population (all patients randomized into the study). As a sensitivity analysis, the per-protocol population will be analyzed (all patients actually taking part in the study). Patients not fulfilling the inclusion criteria will be excluded in a separate sensitivity analysis using the per-protocol set.

Patient characteristics and efficacy parameters (secondary and exploratory endpoints) will be analyzed at baseline and 15 weeks. The values for each respective group will be displayed as mean ± standard deviation or mean ± standard error of the mean. Changes from baseline to 15 weeks will be analyzed by robust Student’s *t*-test (Welch test). Frequency data will be displayed as *n* and %. This data will be analyzed by the chi-square test and McNemar test. By means of correlation and regression analysis, the influence of different factors on the training effect will be investigated. *P*-values < 0.05 will be considered significant.

## Discussion

Increases in pulmonary arterial pressure and pulmonary vascular resistance in HFpEF are related to a higher disease burden and a worse outcome [16, 55]. As treatment possibilities in HFpEF-PH are very limited and non-specific [1, 21, 25, 26], additional pharmacological and non-pharmacological therapeutic strategies are needed.

There are no evidence-based recommendations for the exercise therapy of PH-HFpEF, but taking into consideration the positive impact of exercise training both in PAH and HFpEF, we could expect it to be beneficial in HFpEF-PH as well. However, this hypothesis requires dedicated research, as HFpEF-PH patients differ in hemodynamics and exercise intolerance, compared with HFpEF without PH [56]. The safety, tolerability, and effectiveness of exercising in this specific population were never evaluated so far—most of PH training studies excluded patients with post-capillary PH, and HFpEF trials usually did not focus on right heart and pulmonary circulation [33, 48].

The intervention that we are planning to investigate is a standardized low-intensity rehabilitation program, including exercise and respiratory therapy and mental gait training, which has already demonstrated its safety and effectiveness for PAH and CTEPH patients in several trials, including randomized multicenter study in eleven centers across ten European countries [30, 32, 35, 36, 50, 51].

We believe that randomized controlled multicenter trial, which protocol we are sharing in this article, will add important knowledge about the potential utility of a standardized exercise training program for HFpEF-PH and will be valuable in finding optimal treatment strategies for these severely ill patients.

## Trial status

Protocol version number and date: version 1.0, 5 December 2019.

Start date of recruitment 2023 Q1.

Estimated primary completion date 2025 Q4.

## Supplementary Information

**Additional file 1. **Reporting checklist for protocol of a clinical trial.

## Data Availability

Only researchers that are directly involved in the study will have access to the final dataset.

## Abbreviations

6-MWD: 6-Min walk test distance
CI: Cardiac index
CO: Cardiac output
CpcPH: Combined post- and pre-capillary pulmonary hypertension
CTEPH: Chronic thromboembolic pulmonary hypertension
DLCO: Diffusing capacity for carbon monoxide
ERS: European Respiratory Society
ESC: European Society of Cardiology
FEV1: Forced expiratory volume in the first second
FVC: Forced vital capacity
HADS: Hospital Anxiety and Depression Scale
HF: Heart failure
HFpEF: Heart failure with preserved ejection fraction
HFrEF: Heart failure with reduced ejection fraction
LVEDP: Left ventricular end diastolic pressure
mPAP: Mean pulmonary arterial pressure
MRI: Magnetic resonance imaging
PAH: Pulmonary arterial hypertension
PAP: Pulmonary arterial pressure
PCWP: Pulmonary capillary wedge pressure
PH: Pulmonary hypertension
PH–LHD: Pulmonary hypertension associated with left heart disease
PVR: Pulmonary vascular resistance
QoL: Quality of life
RCT: Randomized controlled trial
RHC: Right heart catheterization
SF-36: 36-Item Short Form Health Survey
SGLT2: Sodium-glucose cotransporter-2
sPAP: Systolic pulmonary artery pressure
VE/VCO_2_: Ventilatory equivalent for carbon dioxide
VE/VCO_2_ slope: Ventilatory equivalent for carbon dioxide
VO_2_: Oxygen uptake
WHO: World Health Organization
WU: Wood units
